# *OPCML* Is a Broad Tumor Suppressor for Multiple Carcinomas and Lymphomas with Frequently Epigenetic Inactivation

**DOI:** 10.1371/journal.pone.0002990

**Published:** 2008-08-20

**Authors:** Yan Cui, Ying Ying, Andrew van Hasselt, Ka Man Ng, Jun Yu, Qian Zhang, Jie Jin, Dingxie Liu, Johng S. Rhim, Sun Young Rha, Myriam Loyo, Anthony T. C. Chan, Gopesh Srivastava, George S. W. Tsao, Grant C. Sellar, Joseph J. Y. Sung, David Sidransky, Qian Tao

**Affiliations:** 1 Cancer Epigenetics Laboratory, State Key Laboratory in Oncology in South China, Sir YK Pao Center for Cancer, Department of Clinical Oncology, Hong Kong Cancer Institute and Li Ka Shing Institute of Health Sciences, Chinese University of Hong Kong, Hong Kong, China; 2 Department of Surgery, Chinese University of Hong Kong, Hong Kong, China; 3 Institute of Digestive Disease, Department of Medicine and Therapeutics, Chinese University of Hong Kong, Hong Kong, China; 4 Department of Urology, Peking University 1st Hospital & Institute of Urology, Peking University Health Science Center, Beijing, China; 5 Johns Hopkins Singapore, Singapore, Singapore; 6 Center for Prostate Disease Research, Uniformed Services University of the Health Sciences, Bethesda, Maryland, United States of America; 7 Yonsei Cancer Center, Yonsei University College of Medicine, Seoul, Korea; 8 Department of Otolaryngology-Head and Neck Surgery, Johns Hopkins School of Medicine, Baltimore, Maryland, United States of America; 9 Department of Pathology, University of Hong Kong, Hong Kong, China; 10 Department of Anatomy, University of Hong Kong, Hong Kong, China; 11 Cancer Research UK Edinburgh Oncology Unit, University of Edinburgh Cancer Research Center, Edinburgh, United Kingdom; Texas Tech University Health Sciences Center, United States of America

## Abstract

**Background:**

Identification of tumor suppressor genes (TSGs) silenced by CpG methylation uncovers the molecular mechanism of tumorigenesis and potential tumor biomarkers. Loss of heterozygosity at 11q25 is common in multiple tumors including nasopharyngeal carcinoma (NPC). *OPCML,* located at 11q25, is one of the downregulated genes we identified through digital expression subtraction.

**Methodology/Principal Findings:**

Semi-quantitative RT-PCR showed frequent *OPCML* silencing in NPC and other common tumors, with no homozygous deletion detected by multiplex differential DNA-PCR. Instead, promoter methylation of *OPCML* was frequently detected in multiple carcinoma cell lines (nasopharyngeal, esophageal, lung, gastric, colon, liver, breast, cervix, prostate), lymphoma cell lines (non-Hodgkin and Hodgkin lymphoma, nasal NK/T-cell lymphoma) and primary tumors, but not in any non-tumor cell line and seldom weakly methylated in normal epithelial tissues. Pharmacological and genetic demethylation restored *OPCML* expression, indicating a direct epigenetic silencing. We further found that *OPCML* is stress-responsive, but this response is epigenetically impaired when its promoter becomes methylated. Ecotopic expression of OPCML led to significant inhibition of both anchorage-dependent and -independent growth of carcinoma cells with endogenous silencing.

**Conclusions/Significance:**

Thus, through functional epigenetics, we identified *OPCML* as a broad tumor suppressor, which is frequently inactivated by methylation in multiple malignancies.

## Introduction

Epigenetic silencing of tumor suppressor genes (TSGs) is frequently involved in tumor development and progression [1]. Aberrant methylation of promoter CpG islands (CGI) represents a major mechanism of this epigenetic inactivation, which leads to the binding of transcription repressors, compressed chromatin, and transcription silencing [2]. Identification of candidate TSGs silenced by promoter methylation thus uncovers the epigenetic mechanism of carcinogenesis and also identifies new epigenetic tumor markers for early cancer detection [3].

Nasopharyngeal carcinoma (NPC) is a prevalent tumor in Southern China and Southeast Asia, particularly in the Cantonese population [4]. Although virtually all NPC has been shown to be strongly associated with Epstein-Barr virus (EBV) infection [5], [6], the molecular mechanism of NPC pathogenesis is still poorly elucidated [4]. Searches for putative TSGs have identified only few candidates, with tumor-specific promoter methylation, such as *BLU* and *RASSF1A* at 3p21 [7], [8], *CADM1/TSLC1* at 11q23.1 [9]; *THY1/CD90* at 11q22.3 [10], *CDH1* at 16q22.1 [11], *RASAL1* [12], *ADAMTS18* and *CDH13* at 16q23 [13], [14]. These limited findings suggest that additional candidate TSGs are yet to be identified for this tumor.

We previously used a new strategy to search for candidate TSGs genome-wide in NPC, through combining Differential Gene Expression Displayer (DGED) analysis with reported loss of heterozygosity (LOH) data of NPC (Liu & Tao, manuscript in preparation). This strategy revealed a number of putative TSGs that were down-regulated in NPC and also located at LOH loci. One of the *in silico* identified genes is *OPCML* (opioid binding protein/cell adhesion molecule-like gene), also known as *OBCAM* (opioid binding cell adhesion molecule), belonging to the IgLON (OPCML, LSAMP, NEGR1 and HNT) family of glycosylphosphatidylinositol (GPI)-anchored cell adhesion molecules that are highly expressed in the nervous system [15]–[18] and involved in cell adhesion and cell-cell recognition [19]. Located at 11q25, *OPCML* was the first IgLON member linked to tumorigenesis. It was initially identified as a TSG for epithelial ovarian cancer, being frequently inactivated by hemizygous deletion and promoter methylation [20]. More recent studies also demonstrated that *OPCML* is highly methylated in lung adenocarcinoma [21] and down-regulated in gastric and brain carcinomas [22], [23], however no study has been reported for NPC and other tumors yet.

We thus systematically studied its alteration in a series of common tumors. As alternative splicing is a feature of *OPCML* [23] and other IgLONs (e.g. *LSAMP*) [24], we first studied its alternative splicing. We then examined its epigenetic inactivation in NPC and multiple other tumors which have not been studied for this gene, including esophageal, lung, gastric, hepatocellular, colorectal, breast, cervical and prostate carcinomas, as well as non-Hodgkin and Hodgkin lymphomas. We further found that *OPCML* is a stress- and p53-responsive gene; however, its stress response is epigenetically disrupted when the promoter becomes methylated. Ectopic expression of *OPCML* in tumor cell lines with endogenous silencing led to strong inhibition of cell colony formation, demonstrating that *OPCML* acts as a broad tumor suppressor.

## Results

### Identification of novel splicing variants of OPCML

**Figure 1:**
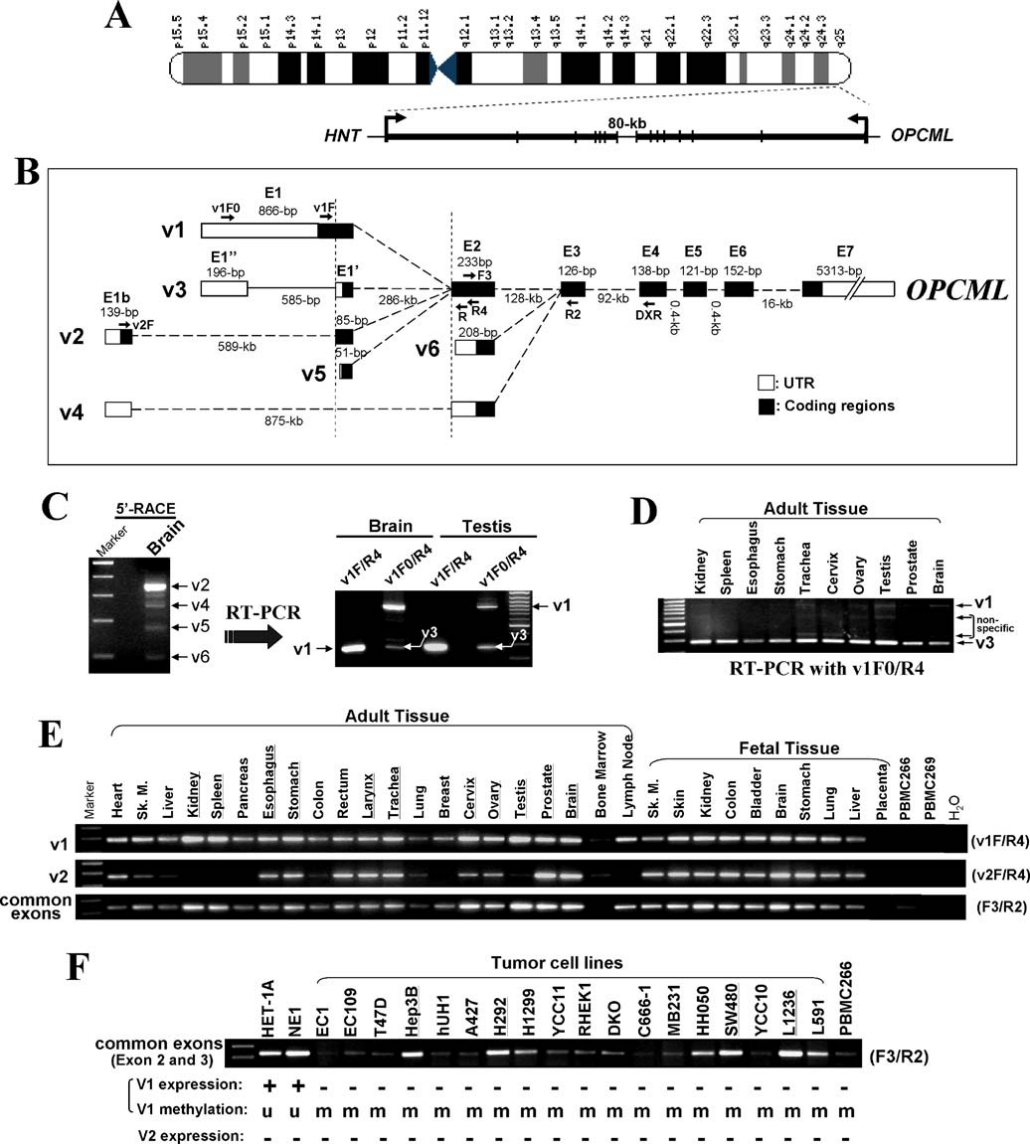
Identification of novel splicing variants of *OPCML* and its expression in normal human tissues. (A) Genomic organization of the 11q25 locus with the two known genes *OPCML* and *HNT*. Transcriptional orientations are shown by curved arrows. (B) Different promoter usage and alternative splicing of *OPCML*. Alternative mRNA transcripts are shown aligned from 5′ to 3′ on a virtual genome. The 5′-end of *OPCML*-v1 assembled by ECgene (Genome Annotation for Alternative Splicing, http://genome.ewha.ac.kr/ECgene/) was adapted to this alignment. (C) Left panel: determination of transcription start sites of *OPCML* transcripts by 5′-RACE. Right panel: expression of *OPCML*-v1 and v3 in brain and testis by semi-quantitative RT-PCR using 5′-RACE product as the template. Primer pair v1F/R4 amplifies one band that is specific to v1. Primer pair v1F0/R4 amplifies two bands corresponding to v1 and v3, respectively. (D) Expression of *OPCML*-v1 and v3 in adult tissues by semi-quantitative RT-PCR. Primer pair v1F0/R4 amplifies two bands corresponding to v1 and v3, respectively. The specific and non-specific bands have been confirmed by direct sequencing. (E) Expression of *OPCML*-v1 and v2 in human normal adult and fetal tissues. Primer pair v1F/R4, v2F/R4 and F3/R2 are specific to the v1-, v2-transcripts, and common exons (exon 2 and 3) of *OPCML* , respectively. Sk.M., skeletal muscle. (F) Possible transcription of *OPCML* from other unidentified alternative promoters. Expression of *OPCML* in normal and tumor cell lines was analyzed by semi-quantitative RT-PCR using primers (F3/R2) specific to common exons (exon 2 and 3) of *OPCML*. Expression of *OPCML*-v1 or v2 is indicated as “+”, while downregulation or silencing is indicated as “−”. *OPCML*-v1 promoter methylation status in each cell line is also shown. M, methylated; U, unmethylated. Transcription of *OPCML* from unknown alternative promoters was found in some tumor cell lines (underlined) where the *OPCML*-v1 promoter is methylated and silenced and v2 expression is also silenced.

*OPCML* contains 7 exons and is transcribed from telomere to centromere (Fig. 1A). Among the four IgLON family members, *OPCML* shares the highest homology to *HNT* that lies approximately 80 kb centromeric to *OPCML* in the opposite orientation. Two alternative splice transcripts of *OPCML*, variant 1 (v1) (NM_002545) and variant 2 (v2) (NM_001012393), were previously identified in human, which differ only in their 5′ exons (Fig. 1B) but encode an identical mature protein [23].

We determined the transcription start sites of *OPCML* using 5′-Rapid Amplification of cDNA Ends (5′-RACE) in human brain and testis RNA. We obtained four PCR products of different sizes (Fig. 1C). Sequence analysis of the major PCR product (EU562296) indicated it as the v2 variant. The 5′ end of v2 was found to be shorter (∼110-bp downstream) than the published data, but its transcription start site matched exactly the DBTSS prediction (DataBase of Transcriptional Start Sites, http://dbtss.hgc.jp/). Three minor splice forms were also identified, designated v4 (EU562298), v5 (EU562299), and v6 (EU562300) (Fig. 1B and 1C left panel). Our 5′-RACE gel electrophoresis failed to reveal a PCR band for the major transcript variant v1 in brain and testis tissues, probably due to the presence of a too large exon 1 (∼1-kb) for v1, which would result in low amplification efficiency. Thus, we performed further RT-PCR using the 5′-RACE product of brain and testis as template and primers specific to v1 (v1F/R4 and v1F0/R4; Fig. 1C right panel), to check whether v1 was expressed in normal tissues. This analysis did confirm the expression of v1 (EU562295) and identified another alternatively spliced variant v3 (EU562297) which is widely expressed in adult tissues (Fig. 1D). Further analysis using primers specific to the common exons (exon 2 and 3) of *OPCML* variants in cell lines without both v1 and v2 transcripts revealed the presence of even more unidentified, alternative promoter usage (Fig. 1F).

### Broad expression of OPCML-v1 and v2 major variants in normal tissues

Previously, *OPCML* was shown to be strongly expressed in brain and normal ovarian epithelia [20]. We further assessed its expression in 33 normal human adult and fetal tissues by semi-quantitative PCR with specific primers targeting the v1, v2, or common exons (exon 2 and 3) (Fig. 1B), respectively. *OPCML*-v1 was widely expressed in all normal adult and fetal tissues except for placenta and peripheral blood mononuclear cells (PBMC), though at varying levels (highly expressed in brain, kidney, spleen, stomach, trachea, testis, cervix, ovary and prostate, and weakly in lung, breast, and bone marrow) (Fig. 1E). Compared to v1, *OPCML*-v2 displayed a more tissue-specific expression pattern in adult tissues, with expression absent or barely detectable in kidney, spleen, pancreas, breast, testis, lung, colon, liver, testis and bone marrow. In contrast to its expression in adult tissues, *OPCML*-v2 was expressed at moderate to high levels in all fetal tissues except for placenta. These results suggest that both v1 and v2 are likely to have important functions in embryonic development.

### Silencing of OPCML by CpG methylation in tumor cell lines

**Figure 2:**
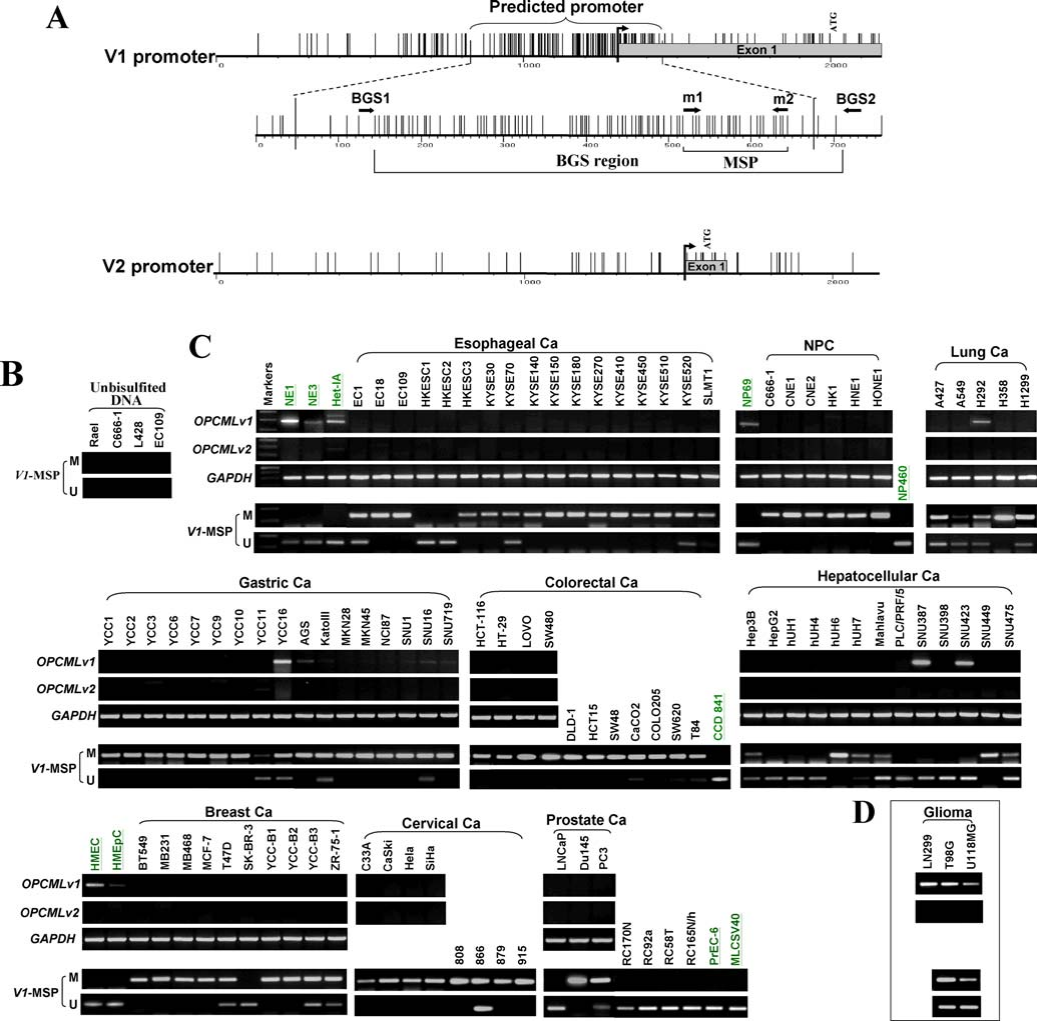
Epigenetic inactivation of *OPCML* in multiple tumor cell lines. (A) Schematic structure of the v1 (NM_002545) CGI, with its exon 1, predicted promoter region, MSP region and BGS region indicated. Each short vertical line represents one CpG site. The v2 promoter is also shown. (B) Validation of the specificity of the MSP system. No signal was detected using the unbisulfited DNA from several tumor cell lines. (C) Representative analyses of *OPCML* v1 and v2 (NM_001012393) expression by semi-quantitative RT-PCR and methylation status of v1-CGI by MSP in tumor cell lines and normal controls. M, methylated; U, unmethylated. Immortalized normal epithelial cell lines (NE1, NE3, Het-1A, NP69, CCD 841, MLCSV40) and normal epithelial cell lines (HMEC, HMEpC and PrEC-6) with underlined names were used as normal controls. RC170N/h and RC165N/h are telomerase-immortalized benign prostate epithelial cell lines, RC92a/h and RC58T/h/SA#4 are telomerase-immortalized prostate tumor derived cell lines. (D) Expression and methylation of *OPCML* in glioma cell lines.

We identified *OPCML* as a down-regulated gene through *in silico* subtraction. We further validated its expression in a large collection of carcinoma and lymphoma cell lines by semi-quantitative RT-PCR. It was found that *OPCML*-v1 expression was dramatically reduced or completely silenced in multiple carcinoma cell lines of nasopharynx, esophagus, breast, cervix, stomach, lung, colon, liver and prostate, as well as in virtually all lymphoma cell lines examined (Fig. 2C and Figure S1), but readily detected in glioma cell lines (Fig. 2D). In contrast, its expression was readily detected in most non-tumor cell lines, including normal mammary (HMEC, HMEpC) and prostate (PrEC-6) epithelial cell line, and immortalized but non-transformed epithelial cell lines (nasopharyngeal, NP69; esophageal, NE1, NE3 and Het-1A; prostate, MLCSV40). Thus, the downregulation of *OPCML*-v1 appeared to be tumor-specific. Notably, expression of *OPCML*-v2 remained undetectable in virtually all cell lines evaluated, including normal cell lines. Given its limited tissue expression pattern and the fact that v2 promoter is not a CpG island with very few CpG sites, the mechanism of *OPCML*-v2 silencing is not pursued further in the current study.

As methylation of promoter CGI is a well-recognized epigenetic mechanism of TSGs silencing [2], we thus examined the potential promoter regions of the 2 major variants (v1 and v2). The *OPCML*-v1 (NM_002545) and v2 (NM_001012393) sequence upstream of their exon 1 was retrieved from the NCBI database and analyzed using promoterinspector (http://www.genomatix.de) and CpG Island Searcher (http://ccnt.hsc.usc.edu/cpgislands2). This analysis predicted a promoter for *OPCML*-v1, located within a typical CGI spanning the published transcription start site of v1, which was also confirmed by our 5′-RACE analysis (Fig. 2A). We did not find any obvious CGI or predicted promoter in the region upstream of the exon 1 of v2, indicating that either the v2 promoter is not a typical one or the published 5′-end sequence of v2 is not complete yet. Thus, we focused on the role of promoter CGI methylation in the silencing of *OPCML*-v1. We first validated that our methylation-specific PCR (MSP) was specific, which did not give any non-specific signal for the unbisulfate DNA (Fig. 2B) and revealed the methylation of *OPCML*-v1 in silenced placenta tissue (Figure S2). Next, v1 promoter methylation was detected in most tumor cell lines with downregulated or silenced expression (6/6 nasopharyngeal, 15/17 esophageal, 5/5 lung, 17/17 gastric, 11/11 colorectal, 6/13 hepatocellular, 9/10 breast, 8/8 cervical and 2/3 prostate carcinoma cell lines, and 20/21 lymphoma cell lines) (Fig. 2C, Figure S1 and Table 1), while no methylation was detected in the eight normal epithelial cell lines, demonstrating that v1 promoter methylation is well correlated with its expression status (Fig. 2C).

**Table 1:** Summary of *OPCML* methylation in cell lines and primary tumors.

Samples	Promoter methylation (%)
*Carcinoma cell lines*	
Nasopharyngeal	5/6 (83%)
Esophageal	15/17 (88%)
Lung	5/5 (100%)
Gastric	16/17 (94%)
Colorectal	11/11 (100%)
Hepatocellular	6/13 (46%)
Breast	9/10 (90%)
Cervical	8/10 (80%)
Prostate	2/3 (67%)
*Lymphoma cell lines*	
Hodgkin's lymphoma (HL)	6/6 (100%)
Burkitt lymphoma (BL)	6/6 (100%)
Diffuse large B-cell lymphoma (DLBCL)	5/5 (100%)
T-cell lymphoma (TL)	1/2 (50%)
NK/T-cell lymphoma (NL)	2/2 (100%)
*Primary tumors*	
Nasopharyngeal Ca	42/43 (98%)
Esophageal Ca	21/32 (66%)
Hepatocellular Ca	4/7(57%)
Gastric Ca	7/11 (64%)
Colorectal Ca	17/18 (94%)
Breast Ca	10/11 (91%)
Cervical Ca	7/8 (88%)
Prostate Ca	0/5
Burkitt lymphoma	10/10 (100%)
Nasal lymphoma	8/9 (89%)
Immortalized *normal epithelial cell lines*	
NP69, NE1, NE3, Het-1A, MLCSV40	0/5
*Normal prostate epithelial cells* (PrEC-6)	0/1
*Normal tissues*	
Normal nasopharynx tissues	3(weak)/9 (33%)
Normal esophageal epithelial tissues	2(weak)/7 (29%)
Normal breast epithelial tissues	1/14 (7%)
Surgical-margin esophageal tissue from esophageal Ca patients	5/32 (16%)
Surgical-margin breast tissue from breast Ca patients	1/4 (25%)

**Figure 3:**
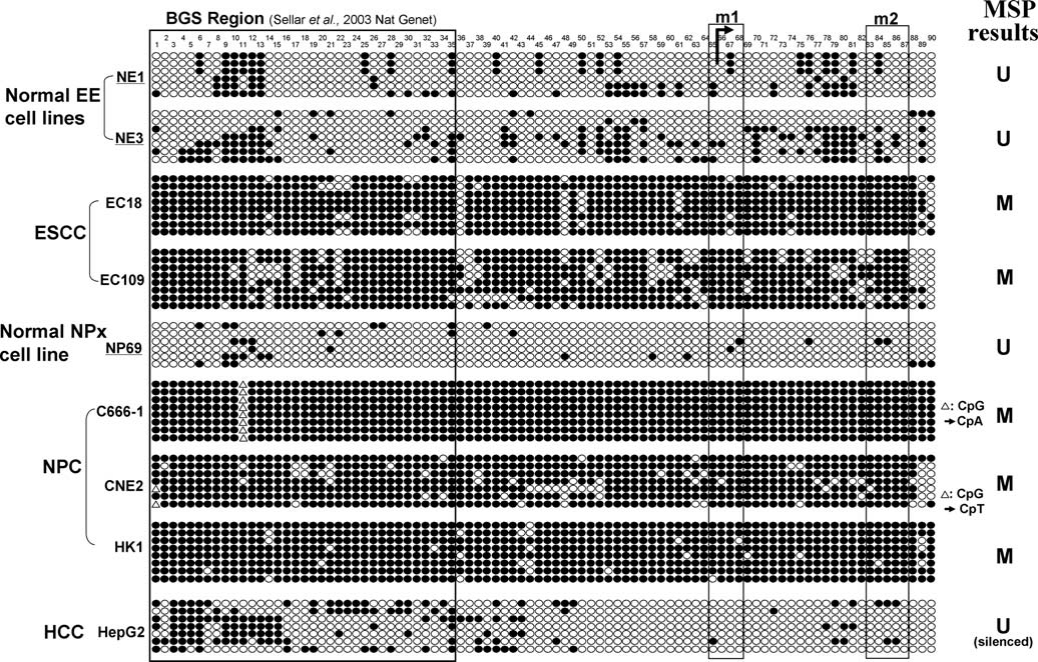
High-resolution methylation analysis of the *OPCML*-v1 promoter by BGS. A region spanning the promoter with 90 CpG sites was analyzed. Each CpG site is shown at the top row as an individual number. Dense methylation of the v1-CGI was found in ESCC (EC18, EC109) and NPC (C666-1, CNE2, HK1) cell lines, but not in normal esophageal (NE1, NE3) and nasopharyngeal epithelial (NP69) cell lines. Five to 8 colonies of cloned BGS-PCR products from each bisulfite-treated DNA sample were sequenced and each is shown as an individual row, representing a single allele of the CGI analyzed. One circle indicates one CpG site. Dark filled or open circles represent methylated or unmethylated CpG sites, respectively. Δ indicates possible variation of a CpG site to the CpA or CpT dinucleotides. The MSP region in this study and the BGS region studied in the previous report [20] are indicated in frames. The rightmost column is the MSP result of each sample.

To further confirm the MSP results and examine the methylation status of the v1 CGI in more detail, we performed high-resolution bisulfite genome sequencing (BGS) analysis of 90 CpG sites within the island, spanning almost the entire predicted promoter. The BGS results were consistent with the MSP analysis, with all the promoter alleles extensively methylated in silenced cell lines and only scattered methylated CpG sites detected in non-tumor cell lines (Fig. 3). These results thus revealed a strong correlation between *OPCML*-v1 promoter methylation and its transcriptional silencing in tumor cell lines.

### Pharmacologic and genetic demethylation restored OPCML-v1 expression

**Figure 4:**
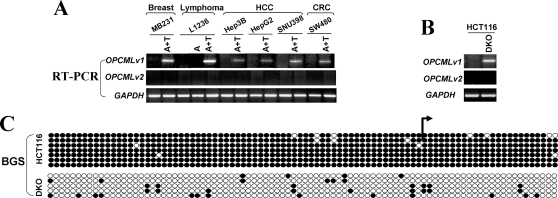
Restorations of *OPCML*-v1 expression by demethylation. (A) Pharmacological demethylation by Aza (A) and TSA (T) induced the expression of *OPCML*-v1 but not v2. *OPCML* expression before and after drug treatment was determined by RT-PCR. (B) Genetic demethylation of the *OPCML*-v1 CGI also activated its expression. *OPCML*-v1 expression in HCT116 cells and HCT116 with double knockout of *DNMT1* and *DNMT 3B* (DKO) are shown. (C) Detailed BGS analysis confirmed the demethylation of the *OPCML*-v1 CGI in DKO cells.

To determine whether methylation directly mediates *OPCML* silencing, carcinoma and lymphoma cell lines (MB231, Hep3B, HepG2, SNU398, SW480 and L1236) were treated with the DNA methyltransferase inhibitor 5-aza-2′-deoxycytidine (Aza), together with or without histone deacetylase inhibitor Trichostatin A (TSA). The treatment resulted in the restoration of *OPCML*-v1 expression in all tumor cell lines (Fig. 4A). *OPCML*-v1 could also be induced in the colorectal cancer cell line HCT116 which is completely methylated for this gene, by genetic demethylation through double knock-out of both DNA methyltransferases *DNMT1* and *DNMT3B* (DKO cell line) [25] (Fig. 4B). Concomitantly, the v1 promoter alleles were almost completely demethylated in DKO cells as confirmed by high-resolution BGS analysis (Fig. 4C), suggesting that the maintenance of *OPCML* methylation is mediated by *DNMT1* and *DNMT3B* together, like other *bona-fide* TSGs that we and others have examined [12], [13], [26]. Interestingly, *OPCML*-v2 could not be activated in any drug treated cell line or DKO cell line (Fig. 4A, 4B), suggesting that the expression of *OPCML*-v2, being tissue-specific, is controlled by other intrinsic mechanism(s), and that its silencing in multiple carcinoma cell lines is controlled by methylation-independent mechanism or, less likely, that its upregulation level is below the limit of detection.

### OPCML downregulation was not due to genetic deletion

**Figure 5:**
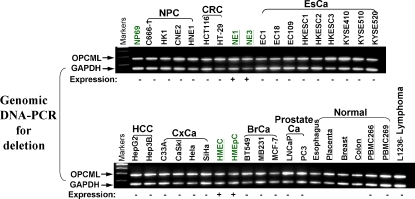
Analysis of homozygous deletion of *OPCML* in multiple carcinoma cell lines and normal controls. The abundance of *OPCML* relative to *GAPDH* was determined by multiplex differential genomic DNA PCR. The expression of *OPCML* in each sample is also shown. +, normal expression; −, downregulated/silenced.

The downregulation of *OPCML* in multiple tumor cell lines might also result from genetic deletion, as it resides in the frequently deleted 11q25 locus. Hemizygous deletion of *OPCML* was also often detected in epithelial ovarian cancer [20]. We thus performed multiplex differential genomic DNA PCR to detect *OPCML* deletion for a region spanning the frequently deleted marker D11S4085 in epithelial ovarian cancer. No homozygous deletion was detected in any silenced tumor cell line (Fig. 5). Furthermore, our high-resolution 1-Mb array comparative genomic hybridization (aCGH) analysis of NPC and ESCC cell lines [12], [26], [27] revealed the hemizygous deletion of *OPCML* in only 2 out of 15 cell lines (data not shown). Thus, downregulation of *OPCML* appears not to be due to genetic deletion, but rather predominantly to epigenetic silencing.

### Frequent methylation of OPCML in multiple primary tumors

**Figure 6:**
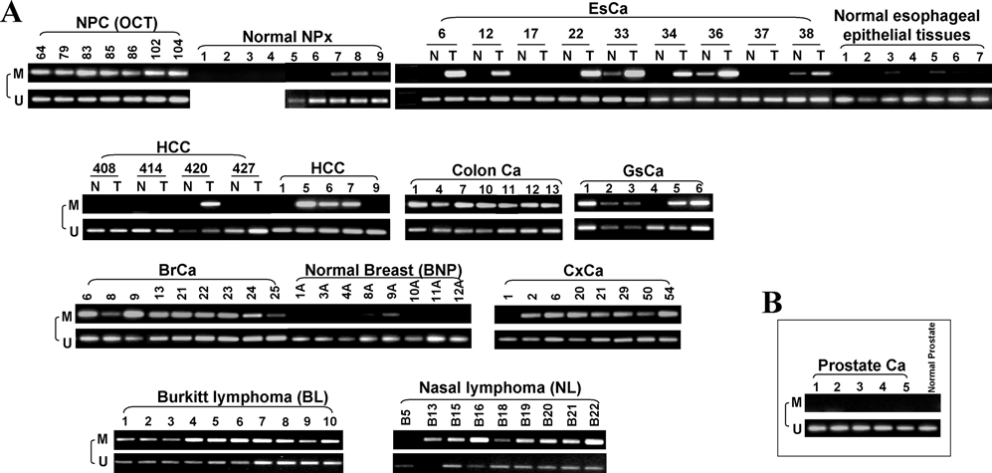
OPCML-v1 was also methylated in different primary tumors. (A) Frequent methylation of the *OPCML*-v1 CGI in multiple primary tumors as analyzed by MSP. M, methylated; U, unmethylated. Representative results are shown. T, tumors; N, paired non-tumor tissues. Good quality of bisulfited DNA samples of normal NPx (NPx1-4) has been confirmed by Q-MSP for beta-actin [44]. (B) In contrast, no methylation was detected in prostate tumors. EsCa, esophageal carcinoma; HCC, hepatocellular carcinoma; GsCa, gastric carcinoma; BrCa, breast carcinoma; CxCa, cervical carcinoma.

We further investigated the *OPCML*-v1 promoter methylation in a large collection of primary tumors, some with corresponding normal tissues as controls (Fig. 6A and Table 1). *OPCML*-v1 methylation was detected in 98% (42/43) of NPC, 66% (21/32) of esophageal, 91% (10/11) of breast, 64% (7/11) of gastric, 94% (17/18) of colorectal, 57% (4/7) of hepatocellular and 88% (7/8) of cervical carcinomas, as well as in 100% (10/10) of Burkitt lymphoma and 89% (8/9) of nasal lymphoma. Methylation was also detected with low frequency in paired surgical marginal tissues from patients with esophageal carcinoma at the rate of 16% (5/32), and with breast carcinoma at the rate of 25% (1/4), which might be due to the presence of small number of tumor cells disseminated into the adjacent non-tumorious region or an early tumor in the adjacent normal regions. Basically no methylation was detected in normal epithelial tissues (nasopharynx, esophagus and breast) except for very weak methylation in three nasopharyngeal, two esophageal and one breast epithelial tissues (Fig. 6A). These results further demonstrated that methylation of *OPCML*-v1 promoter is frequent in multiple tumors. In contrast, no methylation was detected in all five prostate cancer samples (Fig. 6B).

### Promoter methylation disrupted the stress response of OPCML-v1

**Figure 7:**
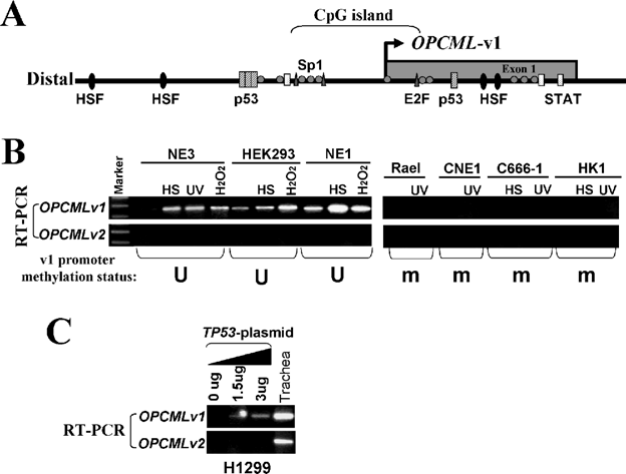
The *OPCML*-v1 promoter is stress- and p53-responsive. (A) Locations of transcription factors (HSF, p53, Sp1, E2F, STAT) binding sites in the promoter are indicated. (B) Up-regulation of *OPCML*-v1 in response to stress treatments is disrupted in tumor cell lines with a methylated promoter. Normal (NE3, HEK293, NE1) and tumor cell lines (Rael, CNE1, C666-1, HK1) were exposed to 42°C heat shock (HS), UV irradiation, or H_2_O_2_ treatments. *OPCML*-v1 promoter methylation status in each cell line is shown at the bottom. M, methylated; U, unmethylated. (C) H1299 cells were transfected with different amounts of pcDNA3.1+/TP53 (gift from Dr. Bert Vogelstein) [27]. Expression of *OPCML*-v1 and v2 was analyzed by semi-quantitative RT-PCR. p53 induced a dosage-dependent upregulation of *OPCML*-v1.

Examination of the *OPCML* promoter revealed multiple HSF and p53 binding elements (MatInspector, http://genomatix.de), indicating that it is a stress- and p53-responsive gene (Fig. 7A). We thus inspected the response of *OPCML* to environmental stress stimuli. We found that the expression of *OPCML*-v1 was dramatically elevated in cell lines with an unmethylated promoter, after exposure to various stresses, such as heat shock, UV irradiation and H_2_O_2_ treatment. On the contrary, this response was significantly decreased or abolished in cell lines with a methylated promoter (Fig. 7B). Interestingly, *OPCML*-v2 was not activated in any stress-treated cell line, indicating that it is not stress-responsive, probably due to its tissue-specific expression feature.

p53 could induce *OPCML* expression in the H1299 cell line with a partially methylated promoter, in a dosage-dependent manner (Fig. 7C). Taken together, these results demonstrated that *OPCML* is a stress-responsive and p53-regulated gene but its stress response is impaired by promoter methylation.

### Ectopic expression of OPCML-v1 inhibited tumor cell clonogenicity

**Figure 8:**
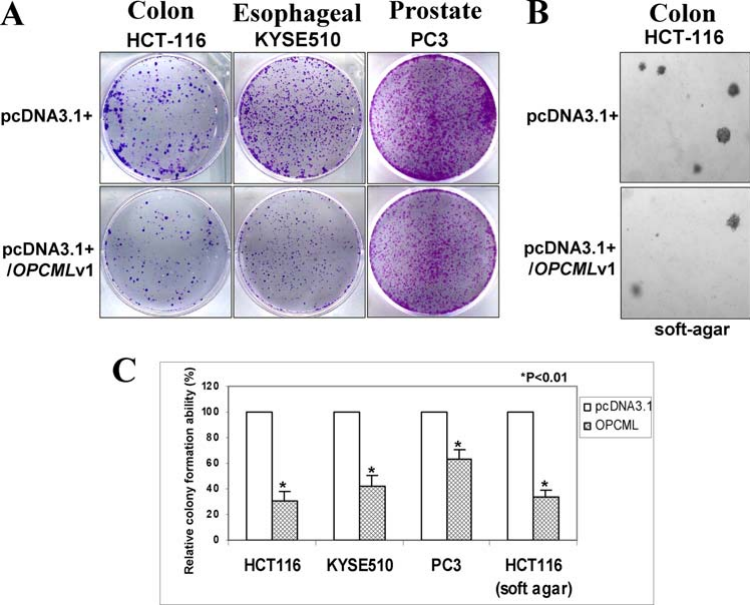
Ectopic expression of OPCML-v1 inhibits tumor cell growth. The effect of ectopic OPCML-v1 expression on carcinoma cell clonogenicity was investigated by monolayer colony formation assay (A) and soft agar assay (B). Cells were transfected with pcDNA3.1+/*OPCML*-v1 or control vector, and selected with G418. (C) Quantitative analyses of colony formation. The numbers of G418-resistant colonies in each vector-transfected control were set to 100%, while OPCML-v1 expressed cells were presented as mean±SD. Three independent experiments were performed in triplicate. The asterisk indicated statistical significant difference (*p*<0.01).

The frequent silencing of *OPCML*-v1 in multiple tumor cell lines and primary tumors but not normal epithelial tissues indicates that *OPCML*-v1 is likely a tumor suppressor. We thus sought to establish whether ectopic expression of OPCML-v1 could inhibit tumor cell clonogenicity. A mammalian expression vector encoding full-length *OPCML*-v1 was transfected into colorectal (HCT116), esophageal (KYSE510) and prostate (PC3) carcinoma cell lines which had completely methylated and silenced endogeous *OPCML*-v1 promoter (Fig. 2C). The colony formation efficiencies of transfected cell line were evaluated by monolayer and soft agar culture. Ectopic expression of OPCML-v1 significantly inhibited the anchorage-dependent growth of three cell lines (down to 30%–60% of vector controls) (Fig. 8A and 8C). Meanwhile, a significant reduction of colony formation efficiencies was observed in anchorage-independent growth of HCT116 cells (down to 30% of vector control) (Fig. 8B and 8C). Thus, *OPCML*-v1 indeed has growth inhibitory activities in tumor cells and can function as a tumor suppressor.

## Discussion

We used a novel approach of combining DGED screening for down-regulated genes with reported LOH data of NPC to search for silenced candidate TSGs genome-wide in NPC and identified *OPCML*. *OPCML* is frequently silenced by promoter methylation rather than genetic deletion in NPC, as well as multiple other carcinomas and lymphomas. We further showed that *OPCML* is a stress-responsive and p53-regulated gene, with the response abrogated when the promoter becomes methylated. In addition, ectopic expression of *OPCML* in carcinoma cells lacking its expression led to dramatic anchorage-dependent and –independent growth inhibition. Thus, our results demonstrate that *OPCML* is a broad functional tumor suppressor that is epigenetically silenced in multiple tumors.

*OPCML* belongs to the IgLON family of immunoglobulin (Ig) domain containing glycosylphosphatidylinositol (GPI)-anchored cell adhesion molecules, which includes OPCML, LSAMP, NEGR1 and HNT. The IgLON proteins are highly conserved between species and are typically composed of three Ig domains tethered to the surface of cell membrane by anchoring of their hydrophobic tails to GPI. Limited knowledge about the functions of IgLONs mainly derives from studies in rat and chick brain, the tissues where they are primarily expressed [17], [28], [29]. In those studies, IgLONs have been suggested to play an important role in cell adhesion and cell-cell recognition, through both homo- and hetero-philic interactions within the family [19]. Recently, it has been proposed that IgLONs function mainly as heterodimers called Diglons [30]. As a cell adhesion molecule, *OPCML* comprises several protein-protein interaction domains, such as three ‘C2’ like Ig domains [31] which are more appropriately classified as ‘I’ set Ig domains [20], commonly found in cell-surface-adhesion and receptor molecules [32]. Through these domains, *OPCML* may bind directly to growth promoting or inhibitory molecules and modulate their functions in tumor cells. Among the IgLON family, *OPCML* was the first member reported to possess tumor suppressor functions in epithelial ovarian cancer, being frequently silenced genetically and epigenetically at the early step of ovarian carcinogenesis [20]. This was followed by another report that another IgLON, *LSAMP*, is also a TSG for renal clear cell carcinoma [33]. Our present study further verifies that *OPCML* can function as a broad TSG and is frequently inactivated epigenetically in multiple carcinomas and lymphomas, including NPC, esophageal, lung, gastric, hepatocellular, colorectal, breast, cervical and prostate carcinomas. *OPCML* probably functions as a tumor suppressor through interacting with other IgLONs to form heterodimeric complex [30] involved in signal transduction. Loss of OPCML reduces the intercellular adhesion and heterodimeric complex formation and thus impairs the corresponding signaling pathways, thereby promoting the progress of carcinogenesis.

*OPCML* shares the highest homology to *HNT* among the four IgLON family members. Notably, the coding region in exon 1 of *OPCML*-v1 and *HNT* is identical, and so is the exon 2 except for only several bases. The first Ig domains of these two proteins share 92% identity, while the second and third Ig domains share 70% and 66% identity, respectively. This raises the possibility that *OPCML* and *HNT* may originate from the same ancestor by gene conversion during evolution. Thus, primers must be cautiously designed for these two genes to avoid cross-amplification with PCR-based techniques.

Our results also reveal that *OPCML* transcripts v1 and v2 have different tissue expression patterns. Whereas *OPCML*-v1 was widely expressed in normal adult tissues, *OPCML*-v2 showed a more tissue-specific expression profile, being highly expressed in few tissues including brain. Previously, a genome-wide searching for the neuron specific silencer REST/NRSF binding sites (RE1/NRSE) revealed that there were three NRSE located at intron 1 of *OPCML*-v2 (http://bioinformatics.leeds.ac.uk/group/online/RE1db/re1db_home.htm), suggesting that v2 may be a more neuron specific transcript. We also identified other novel isoforms of *OPCML* (v3, v4, v5, v6), derived from alternative splicing or promoter usages. Using primers specific to the common exons of *OPCML* transcripts, we found the expression of *OPCML* in several tumor cell lines (Hep3B, H292, SW480, L1236), where the *OPCML*-v1 and v2 were totally silenced (Fig. 1F), indicating transcription of *OPCML* from alternative unknown promoters. Our present study mainly focused on the expression and functional analysis of transcript variant 1, whereas the mechanism of variant 2 silencing was not pursued further. Further studies are needed to characterize these novel splicing variants, their promoter usages and possible biologic functions.

Epigenetic gene silencing is associated with the onset and progression of various cancers [2]. The frequent, predominant epigenetic inactivation of *OPCML* in multiple malignancies points to the importance of this gene in tumorigenesis. *OPCML* is a stress- and p53-responsive gene, but this response was often epigenetically impaired by promoter methylation. We speculate that epigenetic silencing of *OPCML* would impair the cellular protective response to environmental stresses in normal cells, thus promoting the development of cancers. As promoter methylation of *OPCML* was pharmacologically and genetically reversible, pharmacologic demethylation therapy will restore its response to stress and p53. The role of *OPCML* in DNA damage repair, apoptosis and cell cycle arrest with respect to stress response remains to be further investigated. We also noticed that in some cell lines (like HCC), *OPMCL*-v1 was silenced without promoter methylation detected by MSP. It could be that for some cell lines, the methylation is not evenly distributed through the CGI (like HepG2 in Fig. 3) and is thus missed by MSP analysis, or additional alternative mechanism such as histone modification is involved.

In summary, we found that the expression of *OPCML*-v1 (NM_002545), a major transcript of this TSG, is frequently silenced or down-regulated in multiple tumors. This inactivation is due to its promoter methylation, which further impairs its response to environmental stresses. We further demonstrated that *OPCML* acts as a broad tumor suppressor for multiple tumor types. The high incidence of epigenetic inactivation of *OPCML* in NPC and esophageal carcinoma, both prevalent in our locality, indicates that *OPCML* methylation could be an epigenetic biomarker for the molecular diagnosis of these tumors.

## Materials and Methods

### Cell lines, tumor and normal tissue samples

A series of tumor cell lines were studied, including nasopharyngeal-NPC, esophageal, lung, gastric, colorectal, hepatocellular, breast, cervical and prostate carcinomas, glioma, Hodgkin and non-Hodgkin lymphomas, including Burkitt lymphoma (BL), diffuse large B-cell lymphoma (DLBCL), T-cell lymphoma (TL) and NK/T-cell lymphoma (NL) [12], [13], [26]. NP69, an SV40 T-antigen-immortalized nasopharyngeal epithelial cell line with many features of normal nasopharyngeal epithelial cells was used as a ‘normal’ control for NPC [34]. Three immortalized normal esophageal epithelial cell lines (NE1, NE3, Het-1A) [26], [35] were used as ‘normal’ controls for esophageal carcinoma. Colon HCT116 cell lines with double knock-out of DNA methyltransferases (DNMTs): HCT116 DNMT1^−/−^ DNMT3B^−/−^ (DKO) cells (gifts from Dr Bert Vogelstein, Johns Hopkins) were used [25]. Total RNA and DNA were extracted from cell pellets using TRI Reagent (Molecular Research Centre, Cincinnati, OH) as reported previously [36]. Cell lines were treated with Aza (Sigma, St. Louis, MO) and TSA as described previously [26].

Human normal adult and fetal tissue RNA samples were purchased commercially (Stratagene, La Jolla, CA, USA or Millipore Chemicon, Billerica, MA, USA) [26]. Human normal tissue DNA samples were purchased from BioChain Institute (Hayward, CA). DNA samples of normal esophageal epithelial tissues were described previously [37], [38]. DNA samples from various primary carcinomas and their corresponding surgical marginal normal tissues (N), were described previously [7], [12], [27], [36], [39]–[43].

### Digital expression subtraction

We searched for downregulated genes genome-wide through Differential Gene Expression Displayer (DGED) analysis (cDNA DGED and SAGE DGED) (http://cgap.nci.nih.gov). This analysis identified a number of downregulated genes in tumors. The candidate gene list was further filtered with the reported loss of heterozygosity (LOH) data of NPC. Genes located at published LOH regions in NPC were extracted using UCSC genome database (http://genome.ucsc.edu).

### 5′-Rapid Amplification of cDNA Ends (5′-RACE)

We determined the *OPCML* transcription start site using 5′-RACE version 2.0 (Invitrogen). Briefly, the first-strand cDNA was synthesized from brain RNA using primer OPCML-DxR, 5′-TCCAGGTACTCATCCTCACT. Homopolymeric tails were then added to the 3′ends with terminal deoxynucleotidyl transferase. PCR was done using Abridged Anchor Primer and a second gene-specific primer OPCML-R2, 5′-CTGCCAATAGCAAGACACAG. The RACE product was enriched by reamplifying with the Abridged Universal Amplification Primer and OPCML-R, 5′-TATGGACCACTTGTCATTCC, cloned and sequenced.

### Semi-quantitative RT-PCR analysis

Reverse transcription-PCR (RT-PCR) was performed for 36 or 37 cycles with hot-start, using Ampli*Taq* Gold DNA Polymerase (Applied Biosystems, Foster City, CA) and *GAPDH* as a control [36]. RT-PCR primers were designed to span introns to prevent amplification of genomic DNA. Primer sequences are provided in Table S1.

### Bisulfite treatment and promoter methylation analysis

Bisulfite modification of DNA, methylation-specific PCR (MSP) and bisulfite genomic sequencing (BGS) were carried out as previously described [26], [36]. Both MSP and BGS were performed for 40 cycles using Ampli*Taq* Gold with hot-start. MSP primers were tested first for not amplifying any unbisulfited DNA. For BGS, the PCR products were cloned into pCR4-TOPO (Invitrogen, Carlsbad, CA), with 5–8 colonies randomly chosen and sequenced. Primer sequences are shown in Table S1.

### Stress treatments

Heat shock was done as previously described [7], except for an incubation at 42°C for 1 hour with recovery at 37°C for 2 hours. For UV treatment, medium was removed and the flask was turned upside down to face the light source in a UV cross-linker (Amersham Biosciences, Piscataway, NJ). Cells were irradiated for a dose of 70 J/m^2^. After irradiation, fresh medium was added, and the cells were recovered at 37°C for 1 hour and then harvested. For H_2_O_2_ treatment, cells were exposed to 0.5 mM of H_2_O_2_ for 1 hour and then harvested.

### Deletion analysis of OPCML by multiplex PCR

Homozygous deletion of OPCML was examined using multiplex genomic DNA PCR as previously described [7]. Primer sequences are shown in Table S1. The final concentration of *OPCML* and *GAPDH* primers is 0.4 µM and 0.2 µM, respectively. PCR products were analyzed on 1.8% agarose gels.

### Colony formation assays

The full-length *OPCML*-v1 ORF was subcloned from the pcDNA3.1 Zeo/*OPCML* plasmid [20] into pcDNA3.1(+) to generate pcDNA3.1+/*OPCML*-v1. HCT-116, KYSE510 and PC3 cells were seeded at 1×10^5^/well in a 12-well plate and allowed to grow for 24h. Cells were then transiently transfected with 0.5 µg of pcDNA3.1+/*OPCML*-v1 or pcDNA 3.1 vector alone, using Fugene6.0 (Roche, Switzerland). For colony formation assay using monolayer culture, cells were collected and plated in a 6-well plate 48h post-transfection, and selected for 1 to 2 weeks with G418 (0.4mg/ml). Surviving colonies (≥50 cells/colony) were counted after staining with Gentian Voilet (ICM Pharma, Singapore). For colony formation assay using soft agar culture, at 48h post-transfection, cells were suspended in RPMI 1640 containing 0.35% agar, 10% fetal bovine serum and 0.4 mg/ml G418 and layered on RPMI containing 0.5% agar, 10% fetal bovine serum and G418 in a 6-well plate. Colonies were photographed at day 20 post-transfection. All the experiments were performed in triplicate wells for three times. Data were presented as relative colony formation ability±SD. Statistical analysis was carried out by Student's t-test, *p*<0.01 was considered as statistically significant difference.

## Supporting Information

Table S1 PCR primers used in this study.
(0.07 MB DOC)

Figure S1 Expression and methylation of *OPCML* in Hodgkin and non-Hodgkin lymphoma cell lines.
(0.17 MB TIF)

Figure S2 Methylation status of the *OPCML-v1* in multiple normal adult and fetal tissues as analyzed by MSP.
(0.11 MB TIF)

## References

[1] Baylin SB, Ohm JE (2006). Epigenetic gene silencing in cancer - a mechanism for early oncogenic pathway addiction?. Nat Rev Cancer.

[2] Jones PA, Baylin SB (2002). The fundamental role of epigenetic events in cancer.. Nat Rev Genet.

[3] Belinsky SA (2004). Gene-promoter hypermethylation as a biomarker in lung cancer.. Nat Rev Cancer.

[4] Tao Q, Chan AT (2007). Nasopharyngeal carcinoma: molecular pathogenesis and therapeutic developments.. Expert Rev Mol Med.

[5] Raab-Traub N (2002). Epstein-Barr virus in the pathogenesis of NPC.. Semin Cancer Biol.

[6] Tao Q, Young LS, Woodman CB, Murray PG (2006). Epstein-Barr virus (EBV) and its associated human cancers–genetics, epigenetics, pathobiology and novel therapeutics.. Front Biosci.

[7] Qiu GH, Tan LK, Loh KS, Lim CY, Srivastava G (2004). The candidate tumor suppressor gene BLU, located at the commonly deleted region 3p21.3, is an E2F-regulated, stress-responsive gene and inactivated by both epigenetic and genetic mechanisms in nasopharyngeal carcinoma.. Oncogene.

[8] Lo KW, Kwong J, Hui AB, Chan SY, To KF (2001). High frequency of promoter hypermethylation of RASSF1A in nasopharyngeal carcinoma.. Cancer Res.

[9] Lung HL, Cheung AK, Xie D, Cheng Y, Kwong FM (2006). TSLC1 is a tumor suppressor gene associated with metastasis in nasopharyngeal carcinoma.. Cancer Res.

[10] Lung HL, Bangarusamy DK, Xie D, Cheung AK, Cheng Y (2005). THY1 is a candidate tumour suppressor gene with decreased expression in metastatic nasopharyngeal carcinoma.. Oncogene.

[11] Chang HW, Chan A, Kwong DL, Wei WI, Sham JS (2003). Evaluation of hypermethylated tumor suppressor genes as tumor markers in mouth and throat rinsing fluid, nasopharyngeal swab and peripheral blood of nasopharygeal carcinoma patient.. Int J Cancer.

[12] Jin H, Wang X, Ying J, Wong AH, Cui Y (2007). Epigenetic silencing of a Ca(2+)-regulated Ras GTPase-activating protein RASAL defines a new mechanism of Ras activation in human cancers.. Proc Natl Acad Sci U S A.

[13] Jin H, Wang X, Ying J, Wong AH, Li H (2007). Epigenetic identification of ADAMTS18 as a novel 16q23.1 tumor suppressor frequently silenced in esophageal, nasopharyngeal and multiple other carcinomas.. Oncogene.

[14] Sun D, Zhang Z, Van dN, Huang G, Ernberg I (2007). Aberrant methylation of CDH13 gene in nasopharyngeal carcinoma could serve as a potential diagnostic biomarker.. Oral Oncol.

[15] Wilson DJ, Kim DS, Clarke GA, Marshall-Clarke S, Moss DJ (1996). A family of glycoproteins (GP55), which inhibit neurite outgrowth, are members of the Ig superfamily and are related to OBCAM, neurotrimin, LAMP and CEPU-1.. J Cell Sci.

[16] Gil OD, Zanazzi G, Struyk AF, Salzer JL (1998). Neurotrimin mediates bifunctional effects on neurite outgrowth via homophilic and heterophilic interactions.. J Neurosci.

[17] Funatsu N, Miyata S, Kumanogoh H, Shigeta M, Hamada K (1999). Characterization of a novel rat brain glycosylphosphatidylinositol-anchored protein (Kilon), a member of the IgLON cell adhesion molecule family.. J Biol Chem.

[18] Eagleson KL, Pimenta AF, Burns MM, Fairfull LD, Cornuet PK (2003). Distinct domains of the limbic system-associated membrane protein (LAMP) mediate discrete effects on neurite outgrowth.. Mol Cell Neurosci.

[19] McNamee CJ, Reed JE, Howard MR, Lodge AP, Moss DJ (2002). Promotion of neuronal cell adhesion by members of the IgLON family occurs in the absence of either support or modification of neurite outgrowth.. J Neurochem.

[20] Sellar GC, Watt KP, Rabiasz GJ, Stronach EA, Li L (2003). OPCML at 11q25 is epigenetically inactivated and has tumor-suppressor function in epithelial ovarian cancer.. Nat Genet.

[21] Tsou JA, Galler JS, Siegmund KD, Laird PW, Turla S (2007). Identification of a panel of sensitive and specific DNA methylation markers for lung adenocarcinoma.. Mol Cancer.

[22] Wang L, Zhu JS, Song MQ, Chen GQ, Chen JL (2006). Comparison of gene expression profiles between primary tumor and metastatic lesions in gastric cancer patients using laser microdissection and cDNA microarray.. World J Gastroenterol.

[23] Reed JE, Dunn JR, du Plessis DG, Shaw EJ, Reeves P (2007). Expression of cellular adhesion molecule ‘OPCML’ is down-regulated in gliomas and other brain tumours.. Neuropathol Appl Neurobiol.

[24] Pimenta AF, Levitt P (2004). Characterization of the genomic structure of the mouse limbic system-associated membrane protein (Lsamp) gene.. Genomics.

[25] Rhee I, Bachman KE, Park BH, Jair KW, Yen RW (2002). DNMT1 and DNMT3b cooperate to silence genes in human cancer cells.. Nature.

[26] Ying J, Li H, Seng TJ, Langford C, Srivastava G (2006). Functional epigenetics identifies a protocadherin PCDH10 as a candidate tumor suppressor for nasopharyngeal, esophageal and multiple other carcinomas with frequent methylation.. Oncogene.

[27] Seng TJ, Low JS, Li H, Cui Y, Goh HK (2007). The major 8p22 tumor suppressor DLC1 is frequently silenced by methylation in both endemic and sporadic nasopharyngeal, esophageal, and cervical carcinomas, and inhibits tumor cell colony formation.. Oncogene.

[28] Lodge AP, Howard MR, McNamee CJ, Moss DJ (2000). Co-localisation, heterophilic interactions and regulated expression of IgLON family proteins in the chick nervous system.. Mol Brain Res.

[29] Gil OD, Zhang L, Chen S, Ren YQ, Pimenta A (2002). Complementary expression and heterophilic interactions between IgLON family members neurotrimin and LAMP.. J Neurobiol.

[30] Reed J, McNamee C, Rackstraw S, Jenkins J, Moss D (2004). Diglons are heterodimeric proteins composed of IgLON subunits, and Diglon-CO inhibits neurite outgrowth from cerebellar granule cells.. J Cell Sci.

[31] Shark KB, Lee NM (1995). Cloning, sequencing and localization to chromosome 11 of a cDNA encoding a human opioid-binding cell adhesion molecule (OBCAM).. Gene.

[32] Harpaz Y, Chothia C (1994). Many of the immunoglobulin superfamily domains in cell adhesion molecules and surface receptors belong to a new structural set which is close to that containing variable domains.. J Mol Biol.

[33] Chen J, Lui WO, Vos MD, Clark GJ, Takahashi M (2003). The t(1;3) breakpoint-spanning genes LSAMP and NORE1 are involved in clear cell renal cell carcinomas.. Cancer Cell.

[34] Tsao SW, Wang X, Liu Y, Cheung YC, Feng H (2002). Establishment of two immortalized nasopharyngeal epithelial cell lines using SV40 large T and HPV16E6/E7 viral oncogenes.. Biochim Biophys Acta.

[35] Deng W, Tsao SW, Guan XY, Lucas JN, Si HX (2004). Distinct profiles of critically short telomeres are a key determinant of different chromosome aberrations in immortalized human cells: whole-genome evidence from multiple cell lines.. Oncogene.

[36] Tao Q, Huang H, Geiman TM, Lim CY, Fu L (2002). Defective de novo methylation of viral and cellular DNA sequences in ICF syndrome cells.. Hum Mol Genet.

[37] Srivastava G, Wong KY, Chiang AK, Lam KY, Tao Q (2000). Coinfection of multiple strains of Epstein-Barr virus in immunocompetent normal individuals: reassessment of the viral carrier state.. Blood.

[38] Wong ML, Tao Q, Fu L, Wong KY, Qiu GH (2006). Aberrant promoter hypermethylation and silencing of the critical 3p21 tumour suppressor gene, RASSF1A, in Chinese oesophageal squamous cell carcinoma.. Int J Oncol.

[39] Ai L, Tao Q, Zhong S, Fields CR, Kim WJ (2006). Inactivation of Wnt inhibitory factor-1 (WIF1) expression by epigenetic silencing is a common event in breast cancer.. Carcinogenesis.

[40] Tao Q, Robertson KD, Manns A, Hildesheim A, Ambinder RF (1998). The Epstein-Barr virus major latent promoter Qp is constitutively active, hypomethylated, and methylation sensitive.. J Virol.

[41] Tao Q, Swinnen LJ, Yang J, Srivastava G, Robertson KD (1999). Methylation status of the Epstein-Barr virus major latent promoter C in iatrogenic B cell lymphoproliferative disease. Application of PCR-based analysis.. Am J Pathol.

[42] Ying J, Srivastava G, Hsieh WS, Gao Z, Murray P (2005). The stress-responsive gene GADD45G is a functional tumor suppressor, with its response to environmental stresses frequently disrupted epigenetically in multiple tumors.. Clin Cancer Res.

[43] Steenbergen RD, Kramer D, Braakhuis BJ, Stern PL, Verheijen RH (2004). TSLC1 gene silencing in cervical cancer cell lines and cervical neoplasia.. J Natl Cancer Inst.

[44] Hoque MO, Kim MS, Ostrow KL, Liu J, Wisman GB (2008). Genome-wide promoter analysis uncovers portions of the cancer methylome.. Cancer Res.

