# Correction: *OPCML* Is a Broad Tumor Suppressor for Multiple Carcinomas and Lymphomas with Frequently Epigenetic Inactivation

**DOI:** 10.1371/annotation/f394b95b-c731-41a3-b0dc-be25fb6a227c

**Published:** 2008-09-12

**Authors:** Yan Cui, Ying Ying, Andrew van Hasselt, Ka Man Ng, Jun Yu, Qian Zhang, Jie Jin, Dingxie Liu, Johng S. Rhim, Sun Young Rha, Myriam Loyo, Anthony T. C. Chan, Gopesh Srivastava, George S. W. Tsao, Grant C. Sellar, Joseph J. Y. Sung, David Sidransky, Qian Tao

In Table S1, the numerical data in the last two rows appear in the wrong columns. Please view the corrected table here: 
