# Supplementary material for: OPCML Is a Broad Tumor Suppressor for Multiple Carcinomas and Lymphomas with Frequently Epigenetic Inactivation
Source: PLoS One. 2008 Aug 20;3(8):e2990. doi: 10.1371/journal.pone.0002990 (PMC2500176; doi:10.1371/journal.pone.0002990)
Supplement: Table S1 — PCR primers used in this study. (0.07 MB DOC) [file pone.0002990.s001.doc]

**Table S1 PCR primers used in this study**

| PCR | Primer | Sequence | Location | Product size (bp) | PCR  cycles | Annealing temp. (^o^C) |
| --- | --- | --- | --- | --- | --- | --- |
| RT-PCR | OPCML-v1F0 | cccgccttggaactttttgc | Exon 1 | 863 (v1F0/R4)-v1  278 (v1F0/R4)-v3 | 36-37 | 58 |
|  | OPCML-v1F | gggtctgtgggtacctgttc | Exon 1 | 280 (v1F/R4) |  |  |
|  | OPCML-v2F | atgtaccatcctgcctactg | Exon 1b | 263 (v2F/R4) |  |  |
|  | OPCML-F3 | ctcgtgtgatcatcctggt | Exon 2 | 229 (F3/R2) |  |  |
|  | OPCML-R2 | ctgccaatagcaagacacag | Exon 3 |  |  |  |
|  | OPCML-R4 | ggtgtattgaccaggatgat | Exon 2 |  |  |  |
|  | GAPDH55 | atctctgccccctctgctga |  | 302 (55/33) | 25 | 60 |
|  | GAPDH33 | gatgaccttgcccacagcct |  |  |  |  |
| MSP | OPCML-m1 | cgtttagtttttcgtgcgttc | v1 Promoter | 129 (m1/m2) | 40 | 65 |
|  | OPCML-m2 | cgaaaacgcgcaaccgacg | v1 Promoter |  |  |  |
|  | OPCML-u1 | tttgtttagttttttgtgtgtttg | v1 Promoter | 136 (u1/u2) | 40 | 60 |
|  | OPCML-u2 | caaaacaaaaacacacaacaaca | v1 Promoter |  |  |  |
| BGS | OPCML-BGS1 | gtttttttgtaggggaagt | v1 Promoter | 609 (BGS1/BGS2) | 40 | 58 |
|  | OPCML-BGS2 | ttattaaatcacacacataaacaa | v1 Promoter |  |  |  |
| DNA-PCR for deletion  (Mutiplex PCR) | OPCML-F3 | ctcgtgtgatcatcctggt | Exon 2 | 272 (F3/int2R) | 32 | 58 |
|  | OPCML-int2R | agtcaaactctgaattcagtag | Intron 2 |  |  |  |
|  | GAPDH-int7F | gcctcactccttttgcagac |  | 155 (int7F/33) |  |  |
|  | GAPDH33 | gatgaccttgcccacagcct |  |  |  |  |
| 5’ RACE |  |  |  |  |  |  |
| cDNA synthesis | DxR | tccaggtactcatcctcact |  |  |  |  |
| PCR1 | R2 (paired with AAP) | ctgccaatagcaagacacag | 58 | 30 |  |  |
| PCR2 | R (paired with AUAP) | tatggaccacttgtcattcc | 60 | 33 |  |  |
|  |  |  |  |  |  |  |
