# Supplementary material for: Correction: OPCML Is a Broad Tumor Suppressor for Multiple Carcinomas and Lymphomas with Frequently Epigenetic Inactivation
Source: PLoS One. 2008 Sep 12;3(9):10.1371/annotation/f394b95b-c731-41a3-b0dc-be25fb6a227c. doi: 10.1371/annotation/f394b95b-c731-41a3-b0dc-be25fb6a227c (PMC2556499; doi:10.1371/annotation/f394b95b-c731-41a3-b0dc-be25fb6a227c)
Supplement: Supplementary file 1 [file pone.f394b95b-c731-41a3-b0dc-be25fb6a227c.s001.pdf]

Table S1 PCR primers used in this study

PCR	Primer	Sequence	Location	Product size (bp)	PCR cycles	Annealing temp. (^{o}C)
RT-PCR	OPCML-v1F0	cccgccttggaactttttgc	Exon 1	863 (v1F0/R4)-v1<br>278 (v1F0/R4)-v3	36-37	58
	OPCML-v1F	gggtctgtgggtacctgttc	Exon 1	280 (v1F/R4)		
	OPCML-v2F	atgtaccatcctgcctactg	Exon 1b	263 (v2F/R4)		
	OPCML-F3	ctcgtgtgatcatcctggt	Exon 2	229 (F3/R2)		
	OPCML-R2	ctgccaatagcaagacacag	Exon 3			
	OPCML-R4	ggtgtattgaccaggatgat	Exon 2			
	GAPDH55	atctctgccccctctgctga		302 (55/33)	25	60
	GAPDH33	gatgaccttgcccacagcct				
MSP	OPCML-m1	cgtttagtttttcgtgcgttc	v1 Promoter	129 (m1/m2)	40	65
	OPCML-m2	cgaaaacgcgcaaccgacg	v1 Promoter			
	OPCML-u1	tttgtttagttttttgtgtgtttg	v1 Promoter	136 (u1/u2)	40	60
	OPCML-u2	caaaacaaaaacacacaacaaca	v1 Promoter			
BGS	OPCML-BGS1	gtttttttgtaggggaagt	v1 Promoter	609 (BGS1/BGS2)	40	58
	OPCML-BGS2	ttattaaatcacacacataaacaa	v1 Promoter			
DNA-PCR	OPCML-F3	ctcgtgtgatcatcctggt	Exon 2	272 (F3/int2R)	32	58
for deletion	OPCML-int2R	agtcaaactctgaattcagtag	Intron 2			
(Mutiplex	GAPDH-int7F	gcctcactcctttgcagac		155 (int7F/33)		
PCR)	GAPDH33	gatgaccttgcccacagcct				
5' RACE						
cDNA synthesis	DxR	tccaggtactcatcctcact				
PCR1	R2 (paired with AAP)	ctgccaatagcaagacacag			30	58
PCR2	R (paired with AUAP)	tatggaccacttgtcattcc			33	60
